# Radical Transparency to Improve Equity in the Kidney Allocation System

**DOI:** 10.34067/KID.0000000000000327

**Published:** 2023-11-27

**Authors:** S. Ali Husain, Joel T. Adler, Sumit Mohan

**Affiliations:** 1 Department of Medicine, Columbia University Medical Center New York, New York; 2 Department of Surgery and Perioperative Care, Dell Medical School, University of Texas at Austin, Austin, Texas

**Keywords:** kidney transplantation, deceased donor kidney, organ allocation, public policy, transplantation

Trust in health care providers and the health care system in the United States lags behind that of peer nations, with particularly high levels of mistrust among historically disadvantaged groups attributable to past and current injustices committed by the medical community.^1,2^ The negative effects of this mistrust are pervasive, affecting access to care, treatment adherence, and health outcomes. Restoring patient trust in the medical system requires ensuring that efforts to improve equity are accompanied by a clear acknowledgment of existing areas of inequity and transparency regarding the corrective actions to ensure parallel improvements in the perception of a commitment to achieving and maintaining equity in the system.

Large inequities exist in ESKD care despite broad Medicare coverage for patients with ESKD. In the United States, non-White race and lower socioeconomic status are associated with an increased risk of kidney failure, a lower likelihood of transplant referral or waitlisting among those who develop kidney failure, and inferior access to transplantation overall. An additional source of inequity in kidney transplantation is organ allocation. The long-standing national organ shortage requires the equitable and just allocation of kidneys from deceased donors. Accordingly, in the United States, candidate priority for each deceased donor kidney is determined primarily based on accumulated waiting time, with prespecified prioritization for specific criteria, such as pediatric status and allosensitization. For each donated kidney, a prioritized list of candidates is generated, and the kidney is allocated to the highest priority candidate who accepts the organ offer. Despite the objective nature of the assignment of allocation priority, only 29% of randomly surveyed individuals in the United States believe the organ allocation system to be fair, with downstream consequences including a lower likelihood of organ donation among those with the expressed belief that the system is unfair.^3^ An emerging body of literature has unfortunately provided objective evidence validating these concerns at multiple steps of the allocation process (Table 1).

**Table 1:** Steps in the deceased donor kidney transplant system that introduce subjectivity and potential solutions to improving transparency

Subjective Assessment	Addition of Transparency
Getting onto the waitlist	• Public reporting of center-level waitlisting rates and time to waitlisting among patients referred for transplant evaluation • Mandate providing a list of alternative centers for evaluation after a patient is declined for listing
Removal from the waitlist for reasons other than transplant or death	• Public reporting of center-level frequency of waitlist removal, reasons for waitlist removal, and center criteria for waitlist removal • Mandated informing of formal internal delisting criteria (*e.g.*, age cutoffs) at the time of first evaluation
Declined organ offers	• Patient-facing organ offer report containing a list of all organs received on behalf of patient over a prespecified time period (*e.g.*, quarterly), including disposition of each organ
Organs not recovered	• Public reporting of the number of eligible deaths for which organ donation consent was achieved, as well as the number of organs for which donation consent was received but recovery did not occur for each organ procurement organization
Out-of-sequence allocation	• Public reporting of out of sequence organ allocation frequency for each organ procurement organization

Transplant centers have the discretion to decline offers for deceased donor kidneys on behalf of their candidates, resulting in the fact that more than 80% of these donated kidneys are transplanted into recipients who did not have the highest waitlist priority for them.^4^ In these cases, centers are not required to inform candidates of offers that were received and declined on their behalf. Kidneys are also increasingly being allocated out of sequence at the discretion of the Organ Procurement Organizations (OPOs), completely bypassing patients prioritized by the allocation algorithm and thereby not allowing these candidates or their transplant centers to even evaluate offers for organs for which they should have priority.^5^ Transplants centers and OPOs typically cite the need for flexibility to deviate from algorithmic allocation priority to ensure that patients toward the top of the waiting list do not inappropriately receive transplants using organs unfit for them or that improving organ utilization should take precedence even if it means skipping down the allocation list to get to centers willing to accept a given organ in a timely manner. However, the characterization of allocation to candidates not at the top of the waiting list suggests that these decisions exacerbate existing transplant disparities.^6^ In addition, the patient-level consequences of these deviations from allocation rules are real and often catastrophic: 30% of waitlisted kidney transplant candidates with at least 1 prior opportunity for a deceased donor kidney transplant that was declined on their behalf and subsequently transplanted into a candidate with lower allocation priority either die or are removed from the waiting list before ever receiving a transplant.^4^ This is further compounded by the subjective nature of candidate delisting that is both accelerating and confounded by significant center-level practice variation, resulting in many candidates being inappropriately deprived of the opportunity to receive a transplant despite spending years waiting for one.^7^ The absence of any meaningful transparency or accountability for these processes means that transplant centers are not required to inform candidates of offers that are declined on their behalf and provide no meaningful public reporting of waitlisting or delisting criteria while OPOs retain unilateral control over the decision to pursue out of sequence allocation.

The subjectivity of these processes undermines the objective design of the allocation system and exacerbates inequity in the kidney transplant system, and the paternalistic nature of these decisions stands in contrast to the shift toward patient engagement in medicine. Increasing transparency to ensure accountability in each phase of the pathway, from kidney transplant waitlisting to transplantation, is necessary to improve trust in the system by openly acknowledging areas of inequity, allowing patients to see the existing challenges in complex decision making and highlighting the efforts at improvements (Table 1). Introducing transparency into these processes must not only include patient-level reporting, including informing patients of organ offers made to them, but also public-facing initiatives, including engagement of the Organ Procurement and Transplantation Network with patients to create meaningful reports regarding the frequency and reasons for organ offer refusal, deviation from standard allocation, and declined transplant waitlisting.

Real-time transparency and shared decision making may be infeasible for every organ offer given the time constraints, but this should not preclude an overall movement toward increasing the amount of information that is made available to patients through readily implementable first steps, including examples shown in Table 1. These recommendations are feasible with currently available data and would not place an undue burden of workload on center, OPOs, or the Organ Procurement and Transplantation Network because they use data that are already collected. For example, although it would not be practical to inform every patient about each offer declined on his or her behalf, a quarterly patient-facing report of all organ offers could allow patients to understand the cadence and types of offers they have received and stimulate shared decision-making discussions to ensure that organ offer responses align with both patient and center preferences. Similarly, given that most patients prioritize getting placed onto the waiting list and getting a transplant above other factors when choosing a center at which to pursue waitlisting,^8^ allowing patients to understand the outcomes of patients with characteristics similar to them who initiate a transplant evaluation at a given center can help inform this decision.

Such transparency can also act as a counterforce to regulatory pressures that perversely incentivize conservative listing and organ utilization practices, given that transplant center regulations have historically focused on post-transplant outcomes without evaluating transplant access or allocation policy adherence, although these measures may not be sufficient to achieve equity in transplantation alone. Improving transparency is necessary to address inequity in kidney transplantation and restore trust in the transplant system, and patients have a right to information about factors that influence their ability to receive a transplant, especially potentially modifiable factors such as subjective provider preferences and behavior.
